# Mechanistic Origins of Yielding in Hybrid Double-Network Hydrogels

**DOI:** 10.1021/acs.macromol.4c02431

**Published:** 2025-08-06

**Authors:** Vinay Kopnar, Adam O’Connell, Natasha Shirshova, Anders Aufderhorst-Roberts

**Affiliations:** † Department of Physics, 3057 Durham University, Lower Mountjoy, South Rd, Durham DH1 3LE, U.K.; ‡ Polymer Science Platform, Reckitt Benckiser Health Care UK Ltd, Dansom Lane S, Hull HU8 7DS, U.K.; § Department of Engineering, 3057 Durham University, Lower Mountjoy, South Rd, Durham DH1 3LE, U.K.; ∥ School of Engineering, University of Liverpool, Liverpool L69 3GH, U.K.

## Abstract

Hybrid double-network hydrogels comprise transiently and permanently cross-linked polymer networks and exhibit enhanced toughness, arising from a local yielding transition. Here, we examine the precise nature of this yielding transition by constructing a series of hydrogel designs from alginate and polyacrylamide (PAAm) networks, systematically controlling their cross-linking chemistry. Using large amplitude oscillatory shear (LAOS) rheology (LAOS), we show the presence of a hitherto unobserved two-step yielding process. Analysis of individual oscillatory cycles and the use of chaotropic/kosmotropic reagents shows that the first step of yielding is determined by the hydrogen bonding *between* the two polymer networks. These interactions also influence the second step of yielding, which we show is governed by the ionic interactions *within* the alginate network. This work demonstrates that interactions *between* are as crucial as interactions *within* the polymer networks and thereby provides insights into how the yielding in soft composite materials can be identified, adjusted, and controlled.

## Graphical abstract

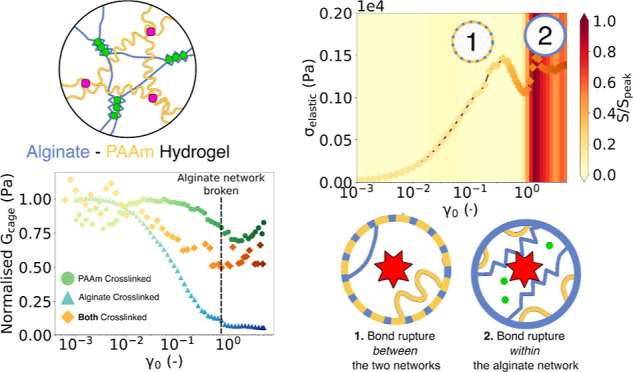

## Introduction

Across nature, almost all living materials are composites and comprise multiple constituent elements that have distinct and complementary mechanical properties. Examples include spider silk, whose high tensile strength arises from the mechanical properties of different structural proteins,^1–3^ and tendons, whose stiffness and compliance strength arise from the combination of elastin and collagen.^4^ Owing to the synergistic effect that combines the best characteristics of the constituents, natural composites exhibit mechanical properties that are inaccessible to single component materials.^5^

For this reason, composite polymeric materials present a useful model system^6–10^ for understanding the mechanical synergy exhibited in living materials.^11^ Of particular note are hybrid double-network hydrogels that contain two interpenetrating polymer networks dispersed in water, one of which is transiently cross-linked through noncovalent interactions and the second of which is permanently cross-linked through covalent bonds.^12^ Hybrid double-network hydrogels undergo fracture at remarkably high strains, and this has been proposed to arise from the ability of the transient cross-links to break under deformation, thereby dissipating energy and preventing the accumulation of elastic stress within the permanently cross-linked network. Experimental^13–15^ and theoretical studies^16–18^ have indicated that this process represents a local yielding mechanism in the hydrogel. Studies have indicated that this yielding occurs at higher applied strains in hybrid double-network hydrogels than in conventional double-network hydrogels that are entirely permanently cross-linked.^19^ However, the precise structural mechanism of this yielding remains a matter of speculation, with potential mechanisms including the dissociation of polymer chains,^19^ the stretching of cross-link junctions,^20^ and the breaking of cross-links.^15^ In addition, the transiently cross-linked network has been proposed to reinforce the permanently cross-linked network, even after yielding,^14^ suggesting direct intermolecular interactions between the two polymer networks.^12,21^ Despite the apparent importance of this yielding process in hybrid double-network hydrogels, it remains challenging to precisely identify the yielding point, to quantify the structural changes that take place during yielding, and to examine how this process relates to the intermolecular interactions between the two polymer networks.

In this work, we address these open questions by probing the mechanical response of a model hybrid double-network hydrogel using oscillatory rheology. Using a well-defined existing protocol,^12^ we construct this hydrogel from alginate and polyacrylamide (PAAm). The alginate/PAAm hybrid double-network hydrogel is an ideal model system for a number of reasons. First, its bulk mechanical properties are well-characterized.^22–24^ Second, the two constituents have distinct and controllable cross-linking chemistries, specifically, the *dN,N*′-methylenebis­(acrylamide) permanent covalent cross-links in the PAAm network and the transient ionic cross-links in the alginate network between Ca^2+^ and α-l-guluronate (G) units of the alginate chains. It is also an excellent model system from the perspective of proving the effect of intermolecular interactions since the two polymers are known to form hydrogen bonds between the carboxyl group of alginate and the amide group of PAAm.^12,25,26^ Using this model system, therefore, allows us to construct a series of hydrogel designs in which either one or both of the cross-links *within* each polymer network are present. Through the use of chaotropic or cosmotropic reagents, we may also either enhance or inhibit the hydrogen bonds that govern the interaction *between* the two polymer networks.

To quantify the yielding mechanism and relate it to structural changes, we use large amplitude oscillatory shear (LAOS) rheology. LAOS is a data-rich rheological method that applies sinusoidal shear strain cycles of increasing amplitude. LAOS reveals information about the rheological response within a given steady-state cycle, while also facilitating the progression of average changes between subsequent steady-state cycles. To study the response of a material in each deformation cycle, we decompose stress into elastic and viscous components using Fourier transform-coupled methods.^27^ Further analysis can then give insights into structural changes, while allowing for the identification of elastic, viscous, and yielding behavior^28^ and visualizing strain-dependent as well as strain-rate dependent phenomena. It also enables comparison of modes of yielding that a hydrogel can undergo^29^ and provides a way to infer microstructural changes within the hydrogel.^30^ It has been used to study the mechanistic origins of the rheological behavior of a wide variety of systems,^28^ including composite hydrogels.^31–33^ However, to our knowledge, the structural and rheological impacts of varying interactions between the two polymer networks in a hybrid double-network hydrogel have yet to be studied. Since LAOS is a novel method to study a hybrid double-network hydrogel, it provides an attractive approach to investigate the role of individual polymer networks on the viscoelastic transformations and yielding behavior.

In this paper, we critically examine the hydrogel designs by utilizing the LAOS framework.

We seek to:

(1) Precisely identify and characterize the yielding transition of the hybrid double-network hydrogel.
(2) Examine how the transient cross-links *within* the alginate network and the permanent cross-links *within* the polyacrylamide (PAAm) influence this yielding transition.
(3) Examine the effect of interactions *between* the two polymer networks on the yielding behavior.

## Materials and Methods

### Hydrogel Preparation

All hybrid double-network hydrogels were prepared from sodium alginate (Sigma-Aldrich, 180947) and polyacrylamide with a 0.11:0.89 mass ratio of the monomers. Initially, a calculated amount of acrylamide (AAm) (Sigma-Aldrich, ≥99%) and alginate were dissolved in the deionized (DI) water to prepare solutions of AAm (5 M) and alginate (5.4% wt/vol). CaCO_3_ was mixed with the required amount of DI water for a total sample volume of 5 mL and a final concentration of 0.045 M. The mixture was sonicated at 25 °C for 20 min. Aliquots from alginate and AAm solutions were mixed to achieve the mass ratio 0.11:0.89. To form the reaction mixture, solutions of *N,N*′-Methylenebis­(acrylamide) (MBA) (Sigma-Aldrich, 99%), a cross-linker for the AAm network, *N,N,N′,N*′-Tetramethylethylenediamine (TEMED) (Sigma-Aldrich, 99%), a cross-linking accelerator, and a freshly prepared solution of ammonium persulfate (APS) (Sigma-Aldrich, ≥98%) were added such that the final concentration of MBA, TEMED, and APS was 0.5 mM, 2.7 mM, and 0.55 mM respectively. The reaction mixture was then purged with nitrogen for 10 s. Next, the suspension of sonicated CaCO_3_ and a freshly prepared solution of glucono-δ-lactone (GDL) (Sigma-Aldrich, ≥99%) was added. To achieve full dissociation of Ca^2+^ ions, the molar ratio between CaCO_3_ to GDL was kept at 1:2. The reaction mixture was then poured into custom-designed molds prepared for high-throughput testing (Figure S1 in Supporting Information) and placed in an oven for 3.5 h at 50 °C after sealing them with vacuum grease and plastic lid. The sealed samples were then stored at room temperature and tested after 21 h. Unless stated otherwise, before testing, all hydrogels in this study were soaked in a solution of CaCO_3_ and GDL with the same concentration as in the reaction mixture for 4.5 h. The dry weight swelling ratio, defined as the ratio of the difference between swollen material weight and dried material weight to the swollen material weight, was determined by drying the hydrogels in a vacuum desiccator until constant weight as determined gravimetrically and found to be 0.75 ± 10%, i.e., the swollen weight is 4 times that of the dry weight, with the hydrogel containing 75 wt % water.

### Hydrogel Design

To illustrate the effect of individual polymer networks’ rheological properties on the rheological behavior of the hybrid double-network hydrogel, we designed two hydrogels containing only one cross-linked network by inhibiting an *intra*polymeric cross-link systematically: Alg+/PAAm- and Alg-/PAAm+. Inhibiting cross-links requires a design strategy that carefully prevents any secondary effects from arising. We synthesized ionically cross-linked Alg+/PAAm- hydrogel by forgoing the chemical cross-linking in the PAAm network, as illustrated in Figure 2a. Similarly, we formed Alg-/PAAm+ hydrogel by excluding the ionic cross-linkers that make up the alginate network while keeping chemical cross-linking in the PAAm intact, as illustrated in Figure 2d. All other reaction mixture components were kept the same as described in section A.

**Figure 1:**
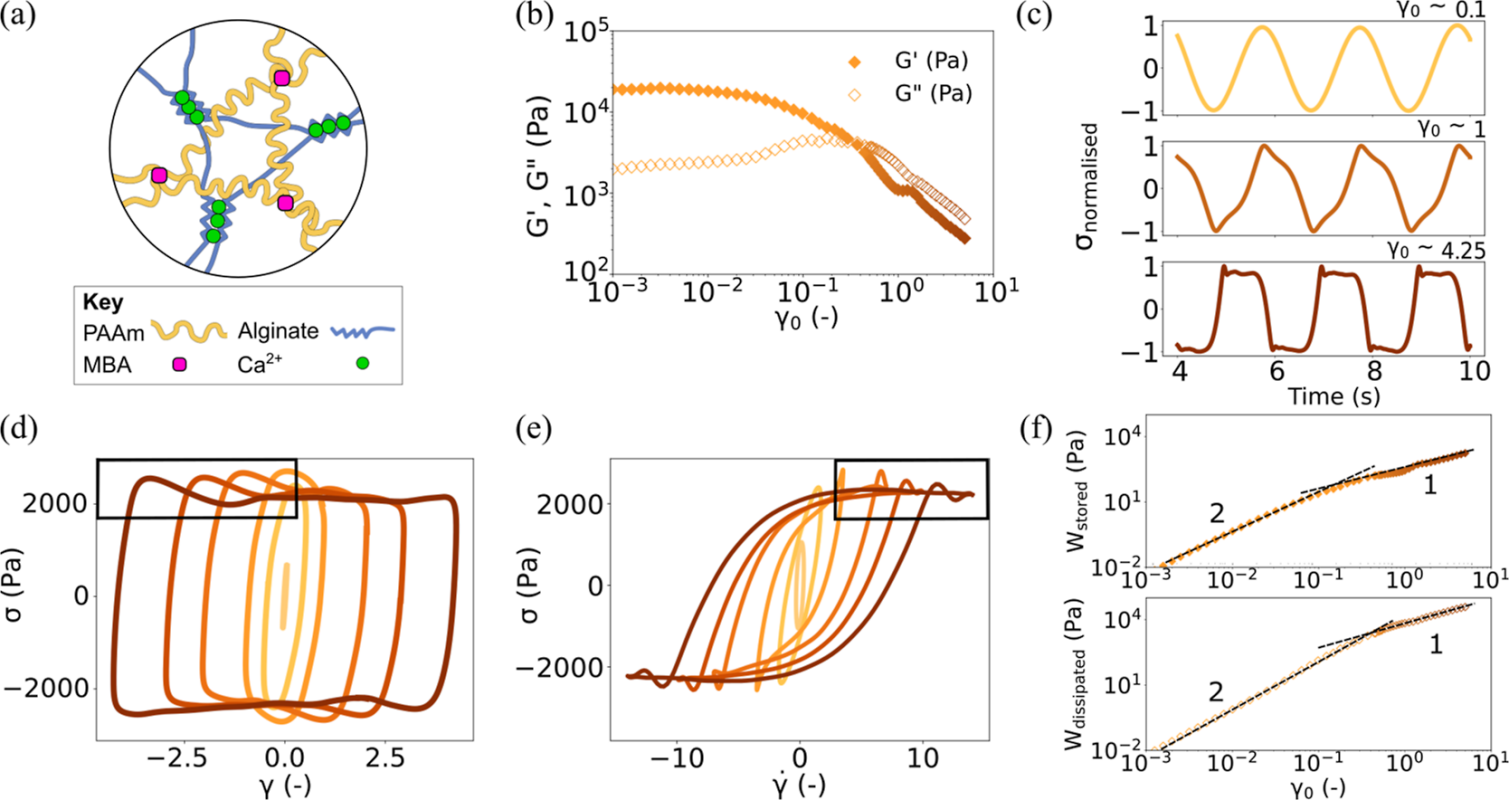
(a) Schematics of the Alg+/PAAm+ hydrogel; (b) evolution of storage (*G*′) and loss (*G*″) moduli with increasing strain amplitude (γ_0_); (c) representation of the normalized stress response (σ_normalized_) to the final cycle of sinusoidal strain with three selected γ_0_ highlighting unambiguous changes with increasing amplitude; (d) elastic LB projections (stress (σ) vs strain (γ)) of the Alg+/PAAm+ hydrogel at selected γ_0_. The black box highlights “stress overshoot” present in projections of high γ_0_; (e) Viscous LB projections (stress (σ) vs strain-rate (γ̇) ) of the Alg+/PAAm+ hydrogel of selected γ_0_. The black box highlights self-intersections present in projections of high γ_0_. For both the projections, the γ_0_ of oscillation increases from light to darker shades; (f) evolution of the energy stored (*W*_stored_) and energy dissipated (*W*_dissipated_) with increasing γ_0_. The dashed lines describe the exponents of the power-law fitted to the adjacent sections on the curves.

**Figure 2:**
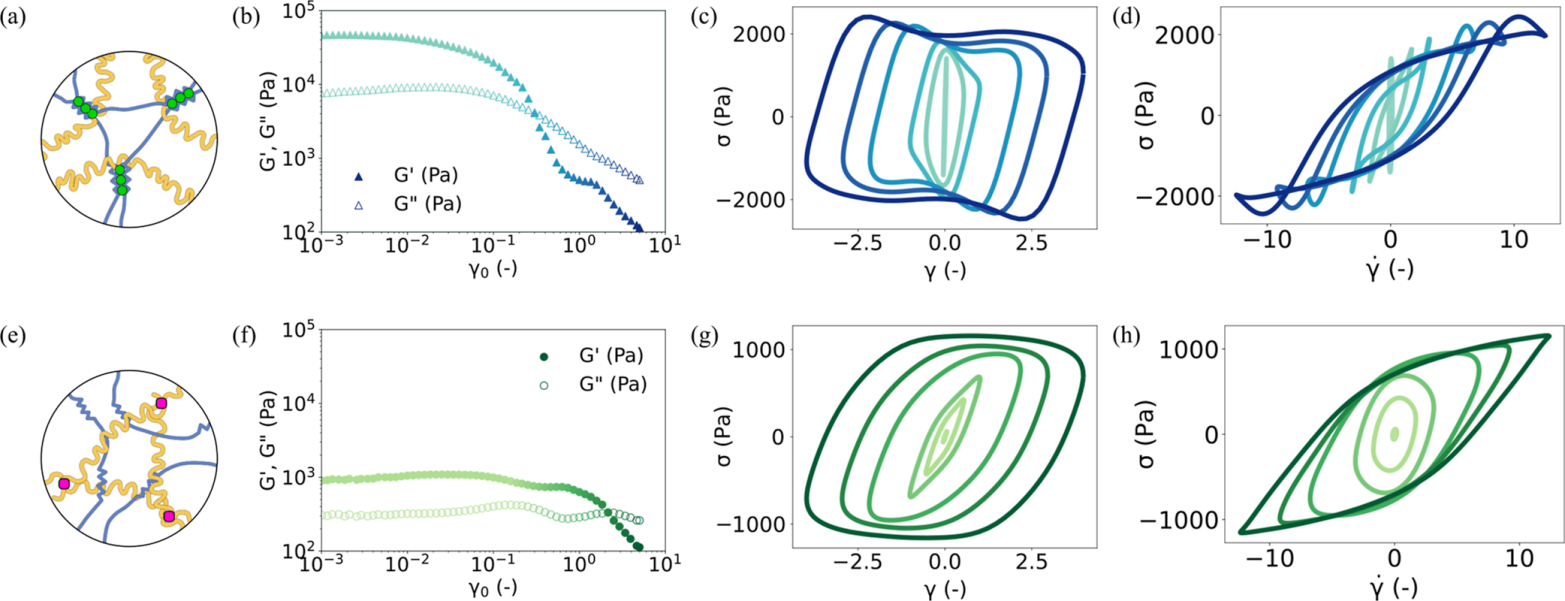
Schematics of structures and behavior under LAOS of the hydrogel designs containing only one network. For Alg+/PAAm- hydrogel, (a) illustrates the schematic of the hydrogel with PAAm chains un-cross-linked; (b) shows the evolution of *G*′ and *G*″ with increasing γ_0_; (c,d) present the elastic and viscous LB projections, respectively. For Alg-/PAAm+ hydrogel, (e) illustrates the schematic of the hydrogel with the alginate chains un-cross-linked; (f) shows the evolution of *G*′ and *G*″ with increasing γ_0_; (g,h) present the elastic and viscous LB projections, respectively. For all the LB projections, the γ_0_ increases from light to darker shades.

Selectively inhibiting one form of polymer cross-linking in this way would have inherently affected the swelling of the hydrogel, which in turn changes the stiffness and other mechanical properties.^34–36^ To maintain a consistent degree of swelling between samples, all hydrogels were soaked in soaking solutions of CaCO_3_ + GDL of the same concentration as in the reaction mixture, until the dry swelling ratio reached 0.75. Measuring the dry swelling ratio for independent samples shows that different hydrogels reach this value over different times, Alg-/PAAm+ hydrogel after 1.5 h, Alg+/PAAm- hydrogel after 2 h, and the hybrid double-network (Alg+/PAAm+) hydrogel after 4.5 h.

To tune *inter*polymeric cross-linking, urea, guanidine hydrochloride (GdHCl), and trimethylamine *N*-oxide (TMAO) with varying concentrations were introduced individually in the soaking solution, and the soaking time was tuned to achieve the same swelling ratio (0.75) as the Alg+/PAAm+ hydrogel. For urea and GdHCl, the soaking time was 4.5 h, while for TMAO, it was 1.5 h.

### Rheology

We used LAOS to study the linear and nonlinear properties of the hydrogel designs. We applied oscillations from 10^–3^ to 5 strain amplitude (γ_0_) at 0.5 Hz frequency using a Netzsch Kinexus Pro stress-controlled rheometer. We monitored how the rheological response changes within a single oscillatory cycle. In LAOS terminology, this is often referred to as intracycle behavior.^27^ We also examined intercycle behavior, which pertains to how behavior changes over the subsequent oscillatory cycles of increasing amplitude. At each amplitude, 10 oscillatory cycles were applied, which is necessary for the strain to reach a steady state strain amplitude, and the response from the final oscillatory cycle was used for analysis. Samples to test were sheared using a 40 mm sandblasted parallel plate unless otherwise stated. A 5 N constant normal force was applied during testing to minimize sample slip. Alg+/PAAm- hydrogel, and the samples reported later, where internetwork cross-links are strengthened, were seen to be prone to slippage. In the case of Alg+/PAAm- hydrogel, an adhesive sandpaper was attached to the top rheometer plate, and a thin layer of super glue was used to adhere the top surface of the sample to the sandpaper. We checked that the use of superglue does not influence sample viscoelasticity by confirming that the frequency spectra of samples with and without superglue overlay within one standard deviation (Figure S2 in Supporting Information). For the samples with strengthened internetwork cross-links, serrated parallel plates were used with a 20 N constant normal force. We used MATLAB to analyze stress–strain curves obtained from testing. To analyze intracycle behavior, particularly, we made use of the MITlaos package,^37^ which assists in extracting relevant parameters from the stress–strain curves.

## Results and Discussion

### Hybrid Double-Network Hydrogels Undergo Continuous Yielding Driven by Transient Cross-Linking

We start by probing the rheological response of a standard Alg+/PAAm+ hydrogel (Figure 1a), where both permanent cross-links and transient cross-links are present and are expected to contribute to the rheological response. We report the dynamic storage (*G*′) and loss (*G*″) moduli as a function of the applied strain amplitude (γ_0_) in Figure 1b. At low γ_0_, we observe a characteristic plateau in both dynamic moduli, signifying the linear viscoelastic (LVE) regime of the hydrogel. Already at a relatively low γ_0_ (>0.01), a decrease in both *G*′ and *G*″ is observed, indicating nonlinear viscoelasticity and signifying the onset of a breakdown in structure.^38^ Within this regime, the dynamic moduli lose their strict physical meaning, but qualitative inferences can still be made. Notably, *G*″ exhibits a characteristic weak overshoot as strain accumulates irreversibly^39^ and, at γ_0_ = 0.3, a crossover is observed in the dynamic moduli, an approximate signature of the material yielding. Beyond this crossover point, we observe a clear shoulder in the value of *G*′ with respect to γ_0_.

Examining the intracycle behavior, in the LVE regime, the stress response (σ­(t)) is initially sinusoidal with a phase shift relative to the strain input (γ­(t)), as seen in Figure 1c. In the Fourier transformation of a sinusoidal signal, one harmonic is typically sufficient to accurately represent the rheological response; however, at higher γ_0_ (>0.1) (Figure 1c), the σ­(*t*) begins to adopt sinusoidal distortions, which indicates the presence of higher order harmonics.^38^ We next assess the emergence, type, and extent of intracycle nonlinear behavior by plotting the rheological response as so-called Lissajous–Bowditch (LB) projections, as shown in Figure 1d,e. Here, the elastic (σ­(t) vs γ­(t)) LB projections of different γ_0_ show that the rheological response adopts an elliptical waveform. Similar elliptical confirmations are seen in the viscous (σ­(t) vs γ̇(t)) LB projections (Figure 1e).

As the sample falls into the non-LVE regime and σ­(t) is distorted, the projections slowly evolve into a curvilinear parallelogram at high strain amplitudes. To illustrate this example, consider the highest amplitude cycles highlighted in Figure 1d (γ_0_ > 1.2). Here, as the strain increases, the intracycle stress is observed to decrease slightly and then subsequently increase to a maximum. This phenomenon is typically referred to as “stress overshoot” and indicates structural rearrangement^40–42^ and the presence of brittle yielding^43^ in the Alg+/PAAm+ hydrogel. This strongly suggests some form of yielding, leading to near-plastic flow. Further supporting evidence of this yielding can be seen in Figure 1e where, within the same range of γ_0_, the viscous LB projections begin to adopt a sigmoidal shape and show self-intersection in high-strain-rate cycles. This self-insertion has been previously linked^44^ to structural rearrangement and, as we observe here, also coincides closely with the stress overshoot.

To examine the origin of this yielding behavior, we consider the time-averaged energy stored and the energy dissipation rate within the Alg+/PAAm+ hydrogel over a single LAOS cycle^45^

1 $$W_{stored}\left(\omega \right)=\frac{1}{4}G^{\prime }\left(\omega \right)\gamma_{0}^{2}$$

2 $$W_{dissipated}\left(\omega \right)=\pi G^{\prime \prime }\left(\omega \right)\gamma_{0}^{2}$$

To examine how the total energy is partitioned as the material yields, we consider the increase in *W*_stored_ and *W*_dissipated_ as γ_0_ increases (Figure 1e). Within the linear regime, both *W*_stored_ and *W*_dissipated_ increase logarithmically with a power law of 2, as expected.^45^ At higher γ_0_ corresponding to the stress overshoot, the power law relationship of both variables levels out to a value of 1. The clear transition between these two regimes is further evidence of yielding as it indicates a lower increase in energy storage after each oscillatory cycle. This transition in energy stored agrees well with the emergence of a stress overshoot observed in Figure 1d and the self-insertion of LB viscous projections in Figure 1e.

A number of inferences can be drawn from this transition. First, the appearance of the stress overshoot in subsequent LAOS cycles indicates that the yielding must be at least partially reversible.^44^ Second, the presence of a shoulder at the yielding transition in Figure 1b has previously been observed in a number of studies of transiently cross-linked hydrogels,^33,46^ although we note that this was not remarked upon by the authors. Finally, the value of *W*_stored_ continues to increase with increasing γ_0_, after yielding, which indicates that the Alg+/PAAm+ hydrogel retains some structural integrity after yielding. Taken together, this indicates that the yielding at γ_0_ > 1.2 is reversible and partial, suggesting a cyclical unbinding and rebinding of the transient Ca^2+^ cross-links, while the permanent chemical cross-links between PAAm likely remain largely intact. We also note the presence of smaller overshoots in σ around near-plastic flow for γ_0_ > 1.2 in Figure 1e. This behavior has been observed in polymer solutions^47^ having bimodal molecular weight distribution and bidisperse polymer melts^48^ and could originate due inherent molecular weight distribution of alginate chains in the alginate network. In the case of alginate network, it is likely that the distribution in alginate chain lengths would lead to a distribution of cross-link unbinding events with respect to strain, with shorter alginate chains unbinding at a low strain and longer alginate chains unbinding at higher strain. This would lead to multiple stress overshoots, as observed in Figure 1e.

### Transient Cross-Links Govern the Rheological Response of the Hybrid Double-Network Hydrogel

To further examine the role of transient cross-links in the rheological response of the Alg+/PAAm+ hydrogel, we now consider the rheological response where one of two intrapolymer cross-links is inhibited. To do this, we synthesize composite hydrogels containing both polymers in which the permanent cross-linking is not present (Alg+/PAAm- hydrogel) or where the transient cross-linking is not present (Alg-/PAAm+ hydrogel), as shown in Figure 2a,e. Performing the identical rheological measurements on both hydrogels, we observe similar rheological responses between the LVE regime of the Alg+/PAAm- hydrogel (Figure 2b) and the Alg+/PAAm+ hydrogel (Figure 1b), in terms of the *G*′ (∼10^4^ Pa), *G*″ (∼10^3^ Pa) and the extent of the LVE regime (γ_0_ < 0.01). Meanwhile, the LVE regime *G*′ and *G*″ of the Alg-/PAAm+ hydrogel are approximately an order of magnitude lower, with a LVE regime that extends significantly further (γ_0_ < 0.1) as seen in Figure 2f. This strongly suggests that the Alg+/PAAm+ hydrogel’s rheological properties in the LVE regime are predominantly governed by the alginate network rather than the PAAm network. The similarity between the LVE regime of the Alg+/PAAm- hydrogel also extends to the nonlinear regime, as shown in Figure 2c. For the Alg+/PAAm- hydrogel, the same distorted elliptical elastic LB projections are observed, as seen in Figure 2c. Notably, at high γ_0_, the Alg+/PAAm- hydrogel exhibits stress overshoot, as seen in the elastic LB projections, in a manner highly reminiscent of the brittle yielding in the Alg+/PAAm+ hydrogel. Similarly, the viscous LB projections of the Alg+/PAAm hydrogel show the same characteristic sigmoidal shape as the Alg+/PAAm+ hydrogels and exhibit the same self-intersection phenomenon at high strain rates (Figure 2d). By contrast, the Alg-/PAAm+ hydrogel manifests an entirely different rheological response. We observe an unambiguous presence of plastic flow in the nonlinear response for γ_0_ > 1.8, which indicates that yielding has taken place. Notably, stress overshoot is entirely absent from the elastic LB projections (Figure 2g), and no self-insertion is observed in the viscous LB projections (Figure 2h), suggesting the presence of ductile yielding.^43^ We therefore conclude that these phenomena directly coincide with the presence of transient cross-links (Figure 1c,d) and are entirely absent when transient cross-links are inhibited (Figure 2g,2h). This provides further validation that the nonlinear response of the Alg+/PAAm+ hydrogel is a direct consequence of the transient cross-linking of the alginate network.

### Stiffening Factor Provides a Reliable Measure of Yielding

Having established the presence of yielding and having correlated this yielding to the unbinding of alginate cross-links, we now seek to provide a quantitative perspective on this yielding, by analyzing each oscillatory cycle using the commonly used method of decomposing σ­(t) into elastic (σ′) and viscous (σ″) components as a series of Chebyshev polynomials of the first kind, *T*_*n*_.^27,28^

3 $$\sigma^{\prime }\left(x:\omega ,\gamma_{0}\right)=\gamma_{0}\sum e_{n}\left(\omega ,\gamma_{0}\right)T_{n}\left(x\right)$$

4 $$\sigma^{\prime \prime }\left(y:\omega ,\gamma_{0}\right)=\dot{\gamma_{0}}\sum v_{n}\left(\omega ,\gamma_{0}\right)T_{n}\left(y\right)$$

where *x* = γ/γ_0_ and *y* = γ̇/γ_0_ depict the normalized strain and strain rate. The coefficients *e* (unit: Pa) and *v* (unit: Pa·s) represent elastic and viscous contributions, respectively. With increasing order, the magnitude of each Chebyshev coefficient decreases monotonically. A physical interpretation of the nonlinearity can be reached by observing the sign of *e*_3_ and *v*_3_ since they determine the concavity of the σ′ and σ″. Changes in these parameters, within and between cycles, reflect changes in the rheological response.^28,49^ Within cycles, this change can be conveniently quantified through the stiffening factor

5 $$S=\left(G_{L}^{\prime }-G_{M}^{\prime }\right)/G_{L}^{\prime }$$

where *G*_*L*_^′^ is a large amplitude secant slope and *G*_*M*_^′^ is a zero amplitude tangent slope, as highlighted in Figure S3 in the Supporting Information. Put simply, *G*_*L*_^′^ probes the rheological response at the maximum strain in a cycle, while *G*_*M*_^′^ characterizes the response at zero strain in a cycle. A value of *S* = 0 indicates *G*_*M*_^′^ = *G*_*L*_^′^ and is therefore indicative of the LVE response; *S* > 0 is taken to indicate intracycle strain stiffening, and *S* < 0 is taken to indicate intracycle strain-softening.

Figure 3a–c shows the stiffening factor *S* with respect to γ_0_ for each Alg+/PAAm+ hydrogel design. In all three hydrogel designs, the value of *S* is close to zero at low values of γ_0_ corresponding to the LVE regime, as expected. At higher γ_0_, all hydrogel designs exhibit a slight temporary decrease in *S,* followed by a sustained increase, signifying intracycle strain stiffening. This strain stiffening corresponds to *e*_3_, the first nonlinear elastic coefficient, taking positive values in all the hydrogel designs (Figure S4 in Supporting Information). The resulting increase in *S* is highest for samples in which alginate is cross-linked (Figure 3b,c) and in both cases reaches a peak at high γ_0_, before decreasing. Meanwhile, when alginate is not cross-linked, stiffening extends to the end of the experimentally accessible range in γ_0_ (Figure 3a). It is likely that this intracycle strain-stiffening arises from the semiflexible nature of the alginate polymer, which results in entropy-driven stiffening as the polymer chains align in the direction of shear.^50^ Notably, a similar behavior has been seen in hydrogels constructed from pectin,^20^ which has a similar molecular structure to alginate. The subsequent decrease in *S* in the presence of alginate cross-linking may be rationalized by the yielding of the alginate network and dissociation of cross-links.^20,46^ The transition between stiffening and yielding is therefore characterized by a peak in *S* and therefore provides a convenient way to clearly identify the yielding of the alginate network.

**Figure 3:**
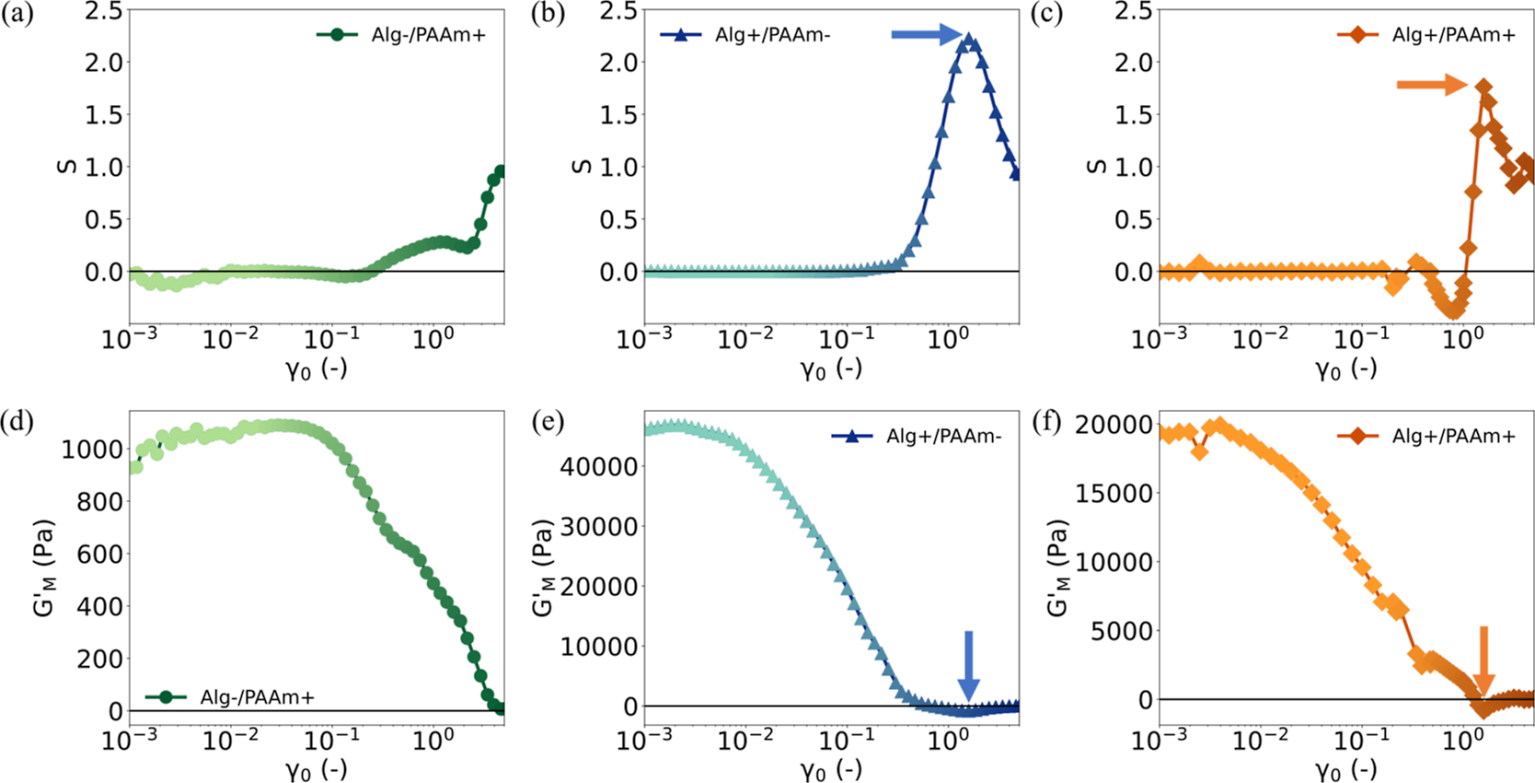
Analysis of intracycle behavior of all the hydrogel designs. For Alg-/PAAm+, Alg+/PAAm-, and the Alg+/PAAm+ hydrogels, (a–c) show the evolution of strain-stiffening parameter (*S*) while (d–f) show the evolution of zero-strain tangent modulus (*G*_*M*_^′^), respectively. The arrows in (b,c) indicate a peak in the *S* which corresponds to *G*_*M*_^′^ taking the most negative values in (e,f).

Another rheological metric that makes the failure evident is the value of *G*_*M*_^′^, as shown for each of the three hydrogel designs in Figure 3d–f. Here, it can be clearly seen that *G*_*M*_^′^ becomes negative when *S* peaks, and furthermore, that maximum in *S* corresponds to a minimum in *G*_*M*_^′^. From an examination of the elastic LB projections in Figures 1d and 2c, it is clear that a negative value of *G*_*M*_^′^ is synonymous with the onset of the negative local slope and stress overshoot. This provides further evidence that the peak in *S* correlates closely with the yielding of the alginate network. Interestingly, in the case of Alg-/PAAm+ hydrogel (Figure 3a), we observe a positive value of *G*_*M*_^′^ across the entire measurement range, which is consistent with the absence of alginate cross-linking. Despite this, some strain stiffening can be seen in Figure 3d, albeit over a longer range of γ_0_ and to a lesser extent.

It should be noted that the conventional amplitude sweep data in Figures 1b, 2b,e would indicate that all hydrogel designs exhibit softening, whereas the positive values of *S* in Figure 3a–c give the paradoxical view of the hydrogels exhibiting strain-stiffening. To resolve this, the elastic stress for the system needs to be examined.^51^ The elastic stress is observed to decrease in successive oscillatory cycles (Figure S5 in Supporting Information), which confirms that the hydrogels undergo gradual softening. We therefore surmise that the parameters *S*, *G*_*M*_^′^, and *G*_*L*_^′^, reflect only the rheological response within a single oscillatory cycle. This makes them beneficial only for characterizing the LB projections, and great care must be taken when using them to make physical interpretations.^52^ Therefore, we may say that all Alg+/PAAm+ hydrogel designs undergo strain stiffening within individual oscillatory cycles but undergo softening after repeated deformation. One final point to support this is that, at large γ_0_, *G*_*M*_^′^ becomes vanishingly close to zero due to the presence of plastic flow at zero-strain as compared to *G*_*L*_^′^, which makes the value of *S* close to 1 inherently.

### PAAm Network Supports the Structure of the Hybrid Double-Network Hydrogel

Having explored the role of the alginate network in the hydrogel’s rheological response, we next examine the contribution of the PAAm network. We have shown that this network remains largely intact across the experimental range of strain amplitudes. We further evidence this by performing forward and reverse sweeps in γ_0_ on Alg-/PAAm+ hydrogel design and verifying that on reversing the γ_0_ sweep direction, *G*′ and *G*″ closely follow the forward sweep values, which highlights that no major structural changes occur in the PAAm network (Figure S6 in Supporting Information). To probe the structural changes in the hydrogel, as the alginate network yields, we consider the local behavior of the network at each stage of the oscillatory shear cycle, as shown in Figure 4. The schematic shows a representative oscillatory cycle carried out on Alg+/PAAm+ hydrogel at γ_0_ ∼ 4.25, an amplitude at which the alginate network yields but the PAAm network remains intact.

**Figure 4:**
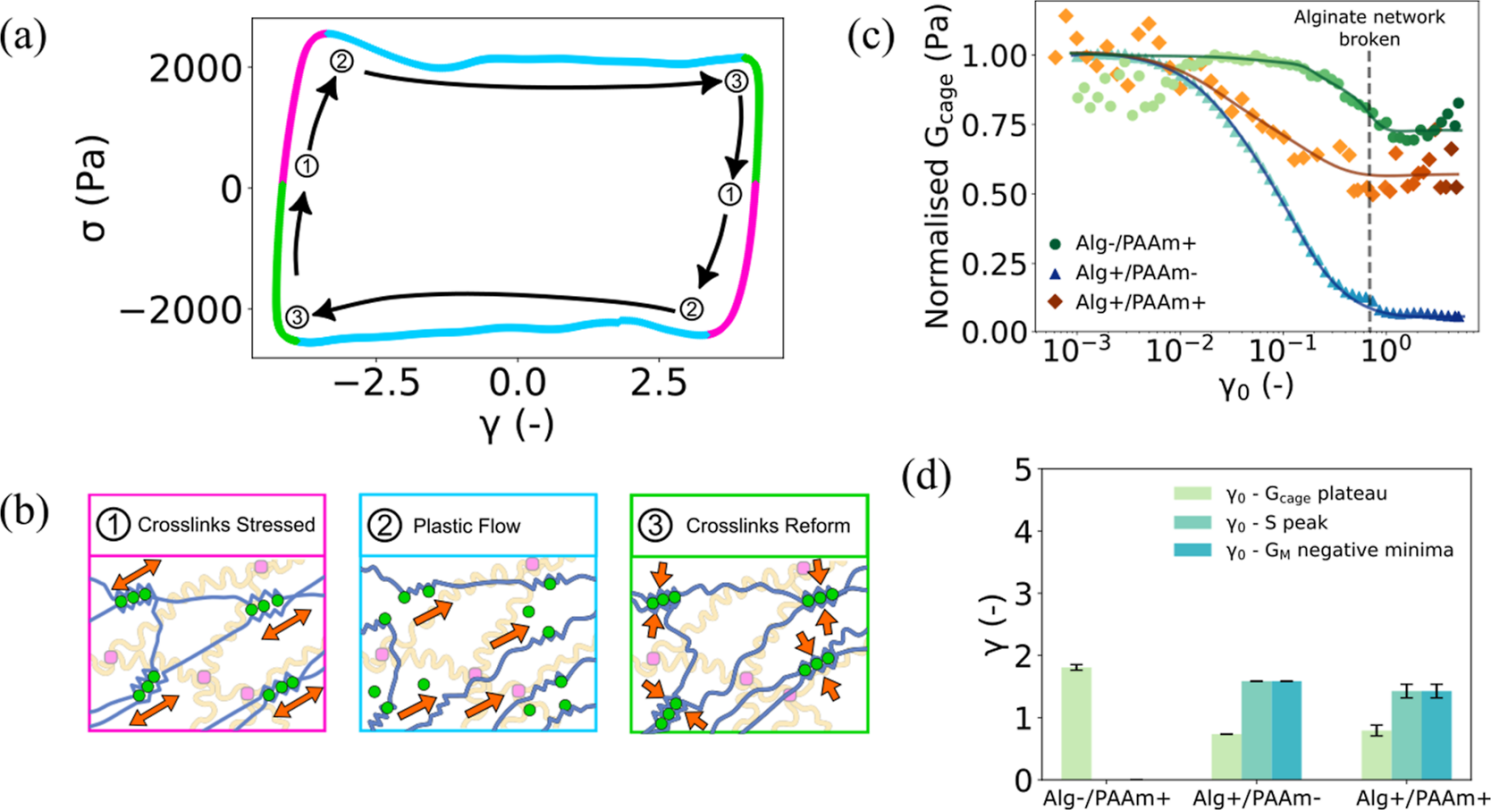
(a) Sequence of physical events that Alg+/PAAm+ hydrogel undergoes in a cycle of large amplitude deformation (γ_0_ ∼ 4.25); (b) schematics of microstructural events in the Alg+/PAAm+ hydrogel corresponding to different highlighted parts on the elastic LB projection presented in (a); (c) evolution of normalized cage modulus (*G*_cage_) for all the hydrogels. The cage modulus has been normalized by the average cage modulus observed in the LVE regime of the hydrogels, and guide-to-eye solid lines are drawn to highlight different features in the evolution. The dashed line highlights the onset of the second plateau in *G*_cage_ at high γ_0_; (d) γ_0_ values for the point at *G*_cage_ starts to plateau at high deformations, the point at which peak in *S* is observed, and the point at negative minima in *G*_*M*_^′^ is observed for all hydrogel designs.

It is important to note that this oscillatory cycle exhibits 180° rotational symmetry around the origin. As with all LAOS waveforms,^44^ this reflects that the underlying structural changes in the material are fully reversible within each cycle. Examining this waveform and beginning at zero shear stress, the hydrogel first exhibits elastic strain as the sample is sheared (highlighted by the pink curve segment; ① → ②). This initial stage reflects the alignment of the alginate polymer chains in the direction of shear as the cross-links are placed under tension (Figure 4b). When the strain is increased further, the stress overshoot is reached (②), at which point the alginate network’s ionic cross-links unbind, leading to a decrease in stress. The breakdown of the alginate network structure induces plastic flow (as highlighted by the blue curve segment; ② → ③). Following this, ③ denotes the point at which the strain rate is zero and γ = ± γ_0_. At this point, the shear stress relaxes (③) as the direction of strain is reversed and the cross-links rebind to cause the alginate network to reform (green curve segment; ③ → ①), likely due to the strong interaction between Ca^2+^ ions and G-units of the alginate chains. The time needed for cross-links rebinding, as observed from the LAOS waveform, which is 1 s (∼ half of the period of oscillation), corresponds well with the noted time scales of reformation ∼ 1.6–10 s as evaluated from stress relaxation behavior of alginate hydrogels.^53,54^

These microstructural changes can be inferred by computing the gradient of the raw σ­(*t*)–γ­(*t*) curve at zero stress. This variable, commonly referred to as the *cage modulus*, *G*_cage_, reflects the rheological response of the hydrogel at mechanical equilibrium within each oscillatory cycle since the elastic and viscous stresses at this point are either zero or momentarily have equal magnitudes with opposite signs.^30^ The evolution of *G*_cage_, normalized by its initial value, with increasing γ_0_, is presented in Figure 4c for all three hydrogel designs. In the LVE regime, *G*_cage_ for all the hydrogels does not vary with γ_0_ and aligns closely with measured values of *G*′ (Figure S7 in Supporting Information). This indicates that no microstructural changes are observed in the LVE regime, as expected. As γ_0_ increases, *G*_cage_ for all the hydrogels begins to decrease, indicating microstructural breakdown.

The nature of this breakdown varies substantially between hydrogel designs. When alginate is cross-linked (Alg+/PAAm- and Alg+/PAAm+ hydrogels), the observed decreases in *G*_cage_ occur at a similar strain amplitude (γ_0_ ∼ 0.01) and at similar initial rates. All hydrogel designs also exhibit a plateau in the normalized *G*_cage_, at high γ_0_. This indicates that there is an upper limit to the elastic energy that is stored by the network^55^ and implies that any further strain imposed on the hydrogel results only in energy dissipation. Where only alginate is cross-linked (Alg+/PAAm- hydrogel), the value of *G*_cage_ reduces by over 90% of its initial value, suggesting a catastrophic microstructural breakdown following yielding.

To understand the role of the PAAm in the yielding process, we compare the evolution of *G*_cage_ between the three hydrogel designs. Comparing Alg+/PAAm- and Alg+/PAAm+ hydrogels, we note that the presence of PAAm cross-linking substantially lessens the structural breakdown since the value of *G*_cage_ reduces to only ∼ 50% of its initial value. From this, we infer that, for the Alg+/PAAm+ hydrogel, once the alginate network breaks, the PAAm network maintains the integrity of the hydrogel and inhibits the macroscopic failure.

Figure 4d summarizes the key features of network failures identified by both modes of analysis presented in the study. The onset of this plateau occurs at a lower γ_0_ than the point at which yielding occurs, as highlighted by the peak in *S* and the negative value of *G*_*M*_^′^ (Figure 3). Thus, we surmise that the structural breakdown reflected by *G*_cage_ can be considered the initial stage of the yielding transition. We note that the point of onset of *G*_cage_ plateau is somewhat subjective, while the peak in *S* can be evaluated precisely. Due to its lack of subjectivity and direct correlation with the yield point, the point of peak in *S* determines the point of yielding of the alginate network in Alg+/PAAm- and Alg+/PAAm+ hydrogels precisely.

The most common general hypothesis for the breakdown of double-network hydrogels under strain posits that the more brittle network (in this case, alginate) fragments into clusters and that these clusters prevent further structural breakdown of the remaining network (in this case, PAAm) by acting as secondary cross-links.^13^ If this were the case here, we would expect that the absence of a cross-linked alginate network would lead to a more dramatic structural breakdown. Instead, we observe the opposite. Where only PAAm is cross-linked (Alg-/PAAm+), the reduction in normalized *G*_cage_ is less severe. Our findings are therefore not consistent with the cluster theory, although it should be emphasized that further structural breakdown might occur at higher γ_0_, beyond the experimentally accessible range of the rheometer. This would be consistent with the ductile nature of the PAAm network.^13,56^ In either case, our data suggest that the mechanical role of the alginate network is to dissipate energy through continuous yielding and reforming, while the role of the PAAm network is to retain the Alg+/PAAm+ hydrogel’s structural integrity and prevent macroscopic failure.

### Hydrogels Containing Transient Cross-Linking Exhibit Two-step Yielding

It should be noted that our use of the peak stiffening factor *S* as a signature of yielding is not a standard metric to quantify yielding. Indeed, there are many approaches to infer a single yield point in the LAOS curves,^57^ including the onset of nonlinearity and the crossover between *G*′ and *G*″. While these definitions are instructive for identifying trends between analyses, they are typically not consistent with one another^58^ and may therefore not offer a true representation of the yield point.^29^ A more reliable approach is to calculate the contribution of elastic stress to the total stress (σ_elastic_ ∼ *G*′γ_0_), from which the yield point may be interpreted as the point at which elastic stress decays.^58–60^ The key advantages of this approach are that it can offer a precise estimation of the yield point, it is less subjective, and it is consistent with conventional steady shear measurements.

To support our claim that the *S* peak is a reliable measure of yielding, we independently quantify the yield point by this method, plotting σ_elastic_ with respect to γ_0_ for all three hydrogel designs in Figure 5. The color bars in the figures represent the values of *S*, extracted from Figure 3, normalized by their maximum values. As expected, for hydrogel designs in which alginate is cross-linked (Figures 5b,c & S8 in Supporting Information), a clear local maximum (labeled (2)) is observed in σ_elastic_ that corresponds directly to the measured peak in *S*.

**Figure 5:**
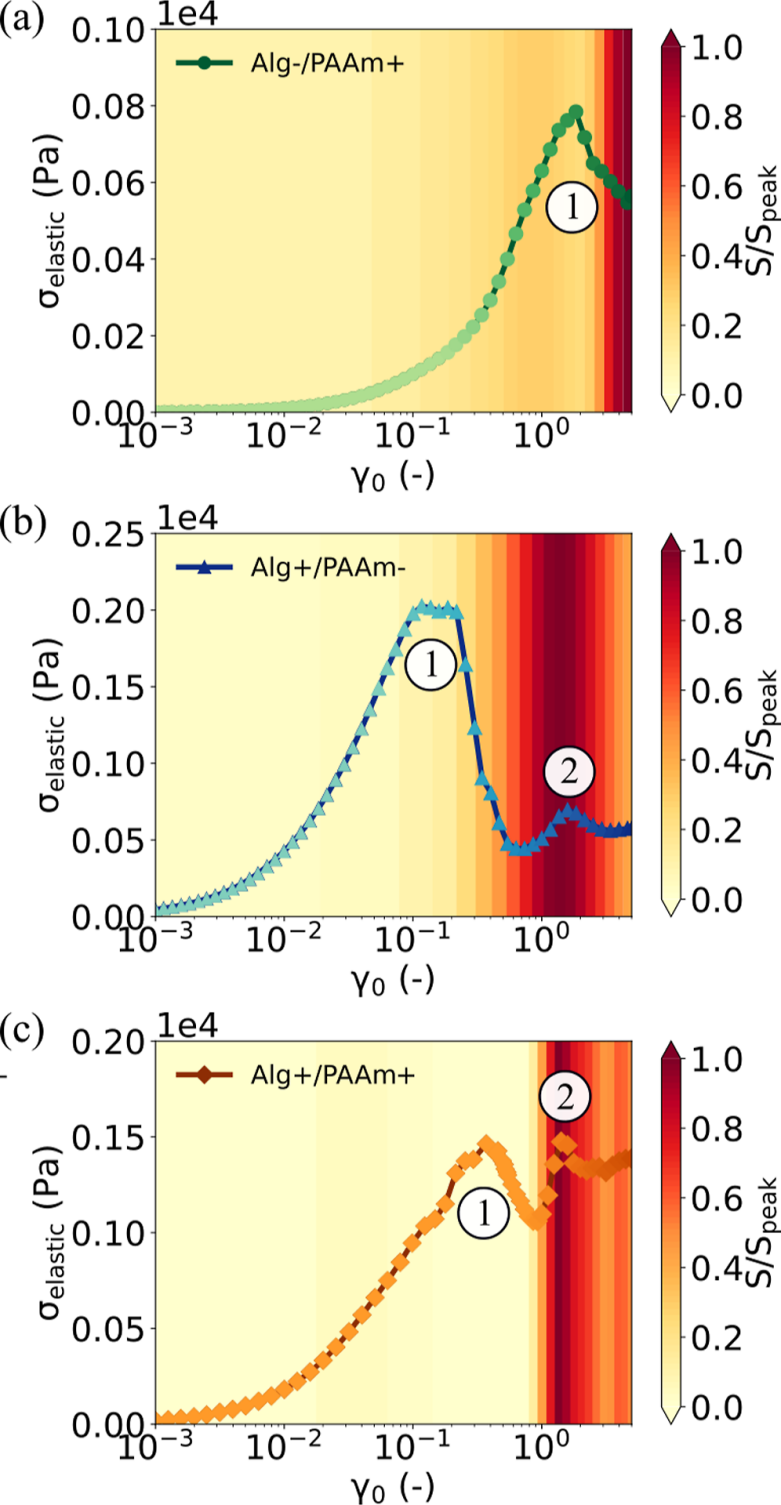
Determination of the yield point. (a–c) Elastic contribution to the total stress (σ_elastic_) experienced by the Alg-/PAAm+, Alg+/PAAm-, and Alg+/PAAm+ hydrogels, respectively. The peaks in σ_elastic_ are numbered as (1) and (2) in the order of occurrence.

Remarkably, we also observe a peak in σ_elastic_ for the Alg-/PAAm+ hydrogel, in which alginate cross-links are not present, and an additional peak for hydrogels containing transient cross-links. In both cases, this peak appears (labeled (1) in Figure 5a–c) at a value of γ_0_ that is approximately 1 order of magnitude below the yield point (2). Examining the relative contribution of the viscous stress (Figure S9 in the Supporting Information), we note that the viscous contributions begin to increase immediately following this initial peak. This therefore indicates that all three hydrogel designs undergo an initial and separate yielding event that is not clearly manifested in the strain amplitude sweep data (Figures 1b and 2b,f) nor in the LB projections (Figures 1d,e and 2c,d,g,h).

The presence of two-step yielding events has been observed for numerous systems^61^ and arises when σ_elastic_ relaxes twice^61^ in a similar manner to that seen in Figure 5b,c. In our system, the second peak (2) coincides with the *S* peaks for hydrogel designs in which alginate is cross-linked, making it evident that the failure in the alginate network triggers yielding. The mechanistic origins of the first peak (1) are less obvious, however. In prior studies of two-step yielding, the emergence of a second peak is generally thought to occur from the presence of a second distinct yielding mechanism.^61^ Examples include cluster breaking in colloidal gels,^62^ cage-breaking in glassy systems,^63^ cage-breaking over different length scales in bidisperse suspensions,^64^ and aggregate remodeling in fibrous networks.^56^ Notably, the emergence of two-step yielding is also often correlated with the presence of an additional component^15^ or a new intermolecular interaction.^56,65^ Considering the components in our hydrogel designs, our interpretation already accounts for the yielding of the alginate network and, furthermore, the observed increase in the energy stored across the experimental range (Figure 1f) indicates that the PAAm network does not yield. Considering possible chemical interactions, the alginate cross-linking and polyacrylamide cross-linking can be discounted for the same reasons. However, there exists a final possible interaction, which is the intermolecular interactions *between* the two polymer networks. Prior spectroscopic studies of similar hydrogel designs have compared the adsorption bands of composite and single-component gels and shown that the combination of PAAm and alginate in hydrogels led to the formation of hydrogen bonds, likely between the –NH_2_ groups of PAAm and the –COO^–^ groups of alginate.^12^ If such hydrogen bonds were present, we would expect them to be weaker than the ionic bonds that cross-link the alginate chains, which is consistent with the presence of the first peak at a lower strain (1) than the second peak (2). It would explain the consistent presence of the additional peak across all three hydrogel designs, across which hydrogen bonding *between* the polymer networks is the only common factor.

### Hydrogen Bonding Provides the Mechanism for Two-step Yielding

Having established hydrogen bonding between the alginate and PAAm polymer networks as a plausible mechanism for the two-step yielding shown in Figure 5, we now test this hypothesis. Investigating the role of hydrogen bonding requires a method to adjust the bond strength. We, therefore, introduce two chaotropic reagents, urea and GdHCl, to our hydrogel designs. Both reagents are expected to disrupt the hydrogen bonds between the two polymer networks of the Alg+/PAAm+ hydrogel, as they preferentially interact with hydrogen binding sites.^66,67^ As a further control, we also introduce the kosmotropic reagent TMAO, which bonds with water molecules and decreases their diffusivity. This ultimately increases the lifetime of the native hydrogen bonds, thereby providing reinforcement,^68^ which results in the increase in *G*′ and *G*″ of the Alg+/PAAm+ hydrogel (Figure S10 in the Supporting Information).

As before, we quantify the yielding point by identifying a peak in the *S* parameter. Figure 6a shows the evolution of *S* with the increase in γ_0_ at different urea concentrations for a hydrogel design in which alginate and PAAm are both cross-linked. Remarkably, the peak in *S* is observed to shift to a lower γ_0_ value as the urea concentration increases, indicating that the hydrogel undergoes yielding more readily when hydrogen bonds are inhibited. Figure 6b depicts the *S* peak shift effect for GdHCl and TMAO alongside urea. Importantly, the addition of GdHCl also results in yielding at a lower strain, which confirms that this phenomenon is a direct result of reduced hydrogen bonding, rather than any other chemical reaction associated individually with urea or GdHCl. Furthermore, the *S* peak is approximately twice as sensitive to GdHCl concentration compared to urea concentration. This is seen by the reduction in the *S* peak from 1.42 to 1.16 in the presence of 1 M GdHCl and from 1.42 to 1.00 in the presence of 2 M urea. The relative ability of GdHCl and urea to disrupt hydrogen bonds in monomeric proteins is generally accepted to be 2:1,^69,70^ providing further evidence that the observed shift in yielding position is linked to the disruption of hydrogen bonds.

**Figure 6:**
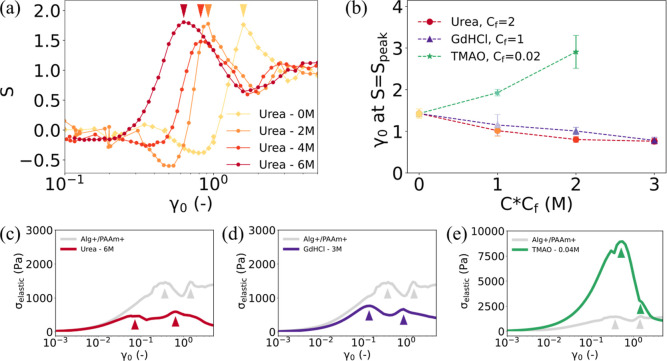
Demonstration of effects of changing the strength of internetwork cross-links. (a) Shift of the peak in *S* to lower values of γ_0_ with the increasing urea concentration in the solution in which Alg+/PAAm+ hydrogel is soaked; (b) evolution of peak shift with increasing urea, GdHCl, and TMAO concentration. A scaling parameter (*C*_f_) is introduced. The actual concentration can be calculated by multiplying the *x*-value of the data point with *C*_f_; (c–e) evolution of σ_elastic_ with increasing γ_0_, for Alg+/PAAm+ hydrogel soaked in 6 M urea, 3 M GdHCl, and 0.04 M TMAO solutions, respectively. The gray curve represents the evolution of σ_elastic_ with an increasing γ_0_ of Alg+/PAAm+ hydrogel, untreated with any of the agents.

By contrast, the reinforcement of hydrogen bonds through the introduction of TMAO increases the strain at which *S* peaks. Here, the *S* peak shifts from 1.42 when no TMAO is present to 1.92 when 0.02 M TMAO is added and to 2.90 when 0.04 M TMAO is added. As a separate measure of yielding, we also plot the evolution of σ_elastic_, examining the original two-step yielding (Figure 5c) in comparison to the equivalent hydrogel design with the inclusion of urea (Figure 6c), GdHCl (Figure 6d), and TMAO (Figure 6e). As expected, the additional peak in σ_elastic_ at low strain is reduced in the presence of urea and GdHCl and increased in the presence of TMAO (Figures 6c–e, & S10 in the Supporting Information). Interestingly, the second peak, which we ascribe to alginate network yielding, is also reduced when hydrogen bonds are inhibited and increased when hydrogen bonds are reinforced. This indicates that the hydrogen bonds play a central role in the rheological response: their presence allows more energy to be stored elastically before the yielding of the alginate network, and their absence limits the ability of the alginate network to store elastic energy.

Overall, our findings indicate a synergy between the two polymer networks that is crucial to understanding the overall rheological response, since the hydrogen bonds between the two polymer networks directly determine the material’s yielding behavior. Based on these findings, it appears that the presence of hydrogen bonds influences the partitioning of elastic energy between the two polymer networks. In the presence of hydrogen bonds, the energy is stored in both polymer networks, and a yielding of the hydrogen bonds is observed before the yielding of the alginate network (Figure 5a). When hydrogen bonds are inhibited, the energy is stored predominantly in the alginate network, such that the mechanical load is not shared between the polymer networks but rather is applied to the alginate polymer chains, leading to the alginate network yielding at lower strain (Figure 6a). Conversely, the reinforcement of the hydrogen bonds enhances the propensity of the two polymer networks to share elastic stress, and the hydrogel undergoes yielding at significantly higher strains.

## Conclusion

We have carried out a systematic synthesis of different hydrogel designs constructed from interpenetrating polymer networks of alginate and PAAm polymer chains and thereby identified the mechanistic origins of their yielding using LAOS rheology. This systematic approach is beneficial, particularly as it allows for direct comparison of mechanical behavior with network structural parameters, and we believe it may be extended to study a plethora of other polymeric composites.

The model system examined here undergoes a process of two-step yielding when the strain amplitude is increased, provided that the alginate network is cross-linked. This is among the very few polymeric hydrogels that have been shown to exhibit the phenomena;^15,56^ however, this may reflect the fact that very few such rheological studies have been carried out on composite hydrogels and, as we argue, probable signatures of two-step yielding may have been overlooked.^33,46^ We believe the quantification of this two-step yielding may present a powerful approach to identifying how polymeric composites undergo yielding. In this study, we show that the first step of this yielding is governed by the breakage of hydrogen bonds between the two polymer networks, while the second step reflects the unbinding of ionic cross-links between the alginate chains. Significantly, both yielding steps are shown to be reversible, as evidenced by the presence of repeated stress overshoot signatures and self-insertion in the LB projections (Figure 1). Our data indicate that, provided both polymer networks are cross-linked, the rebinding of these cross-links occurs within each oscillatory cycle (2 s), indicating that the hydrogels are able to rapidly self-repair. This provides a potential mechanism for the previously reported ability of such hydrogels to dissipate energy under strain^19^ and recover after large deformations.^12^ We have also shown that the alginate network determines the rheological properties in the LVE regime while the largely intact presence of the PAAm network (Figure 4c) indicates its role in maintaining the microstructure of the Alg+/PAAm+ hydrogel after the alginate network ruptures. To our knowledge, this is the first direct rheological evidence to support the hypothesis that a softer and ductile network prevents catastrophic failure in a double-network hydrogel by acting as a sacrificial network.^13^

Our results also highlight a new distinct approach in which the yielding of one network within a composite hydrogel may be identified through the identification of a peak in the stiffening factor *S*. This reflects the semiflexible nature of the alginate chains, which exhibit entropic stiffening as the chains are placed under tension when sheared.^50^ Meanwhile, the flexible nature of the PAAm chains means they do not exhibit stiffening, providing a convenient approach to identify the yielding as the point at which the mechanical load transfers from the semiflexible alginate network to the flexible PAAm network. Remarkably, we show that this yield point is sensitive to the hydrogen bonds that govern the interactions between the two polymer networks. Adding chaotropic reagents that weaken the strength of the hydrogen bonds results in hydrogels that yield at lower γ_0_ while cosmotropic reagents are able to delay the yielding of the alginate network. It will be interesting to examine whether similar trends in yielding emerge from other composite hydrogels, which include semiflexible networks such as agar^19^ or collagen.^71^

Overall, we have combined a systematic synthesis approach with the quantitative analysis of oscillatory rheology data through LAOS rheology. Through this approach, we have found that it is possible to present a nuanced and detailed analysis of rheological behavior and underlying microstructural changes in composite hydrogels. From the perspective of polymer composites, we have shown that cross-links *between* individual polymer networks are as important as the cross-links *within* each network. Thus, controlling the internetwork cross-linking density or strength can be considered as a design parameter for composites that can more closely mimic the mechanical properties of natural tissues.

## Supplementary Material



## Notes

Design of molds and data from the experiments (PDF)
