# Supplementary material for: Mechanistic Origins of Yielding in Hybrid Double-Network Hydrogels
Source: Macromolecules. 2025 Aug 6;58(16):8610–21. doi: 10.1021/acs.macromol.4c02431 (PMC12392726; doi:10.1021/acs.macromol.4c02431)
Supplement: Supplementary file 1 [file ma4c02431_si_001.pdf]

Supporting Information for

Mechanistic Origins of Yielding in Hybrid Double Network Hydrogels

Vinay Kopnar, Adam O'Connell, Natasha Shirshova, and Anders Aufderhorst-Roberts

S1. THE CUSTOM-DESIGNED MOULD

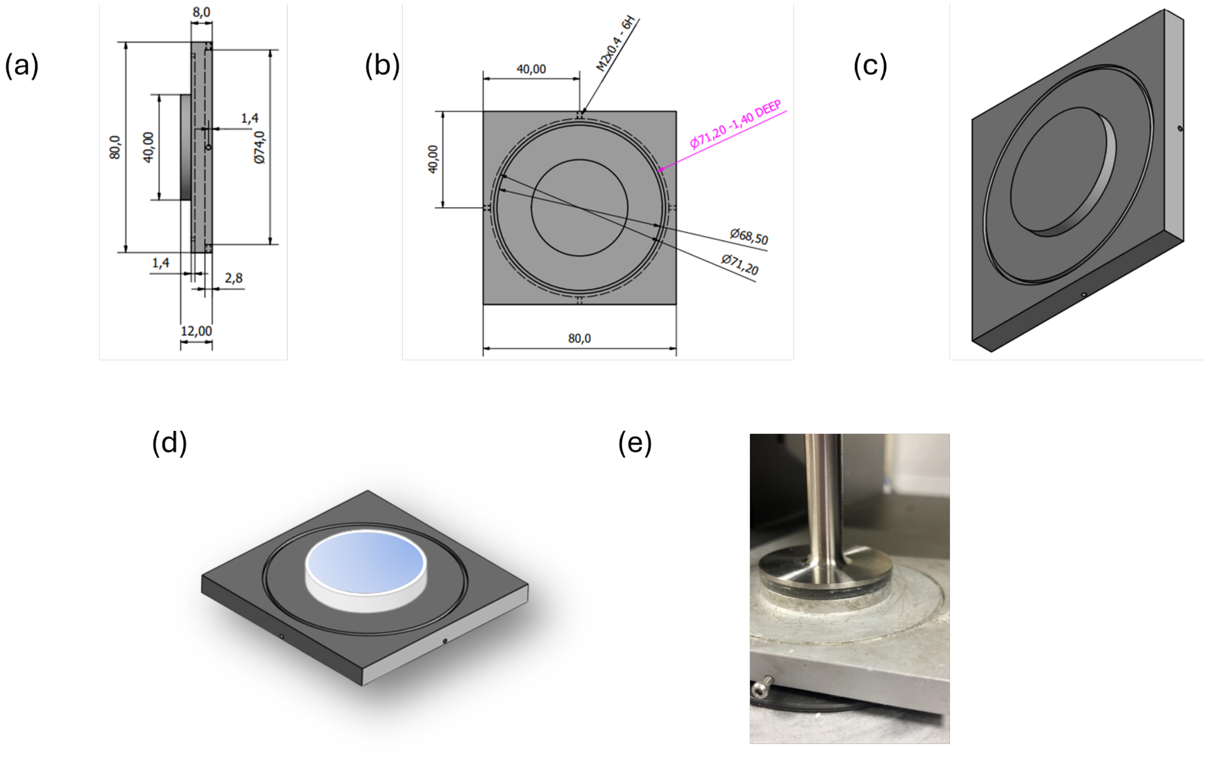

FIG. S1. Custom-designed mould. (a) Dimensions of the mould from the side view. Hydrogels sit on the raised-up portion with 40mm diameter and 4mm height; (b) Dimensions of the mould from the top view. Mould had 4 slots for screws that hold the mould tightly on the bottom plate of the rheometer; (c) Schematic of the mould. The top surface of the raised part was sandblasted; (d) A Teflon sleeve with an inner diameter of 40mm was inserted around the raised metallic part of the mould to contain the liquid reaction mixture. The height of the sleeve was 6mm which meant that the height of the hydrogels was 2mm; (e) Mould under the rheometer top plate during testing

S2. INVESTIGATING THE EFFECT OF SANDPAPER

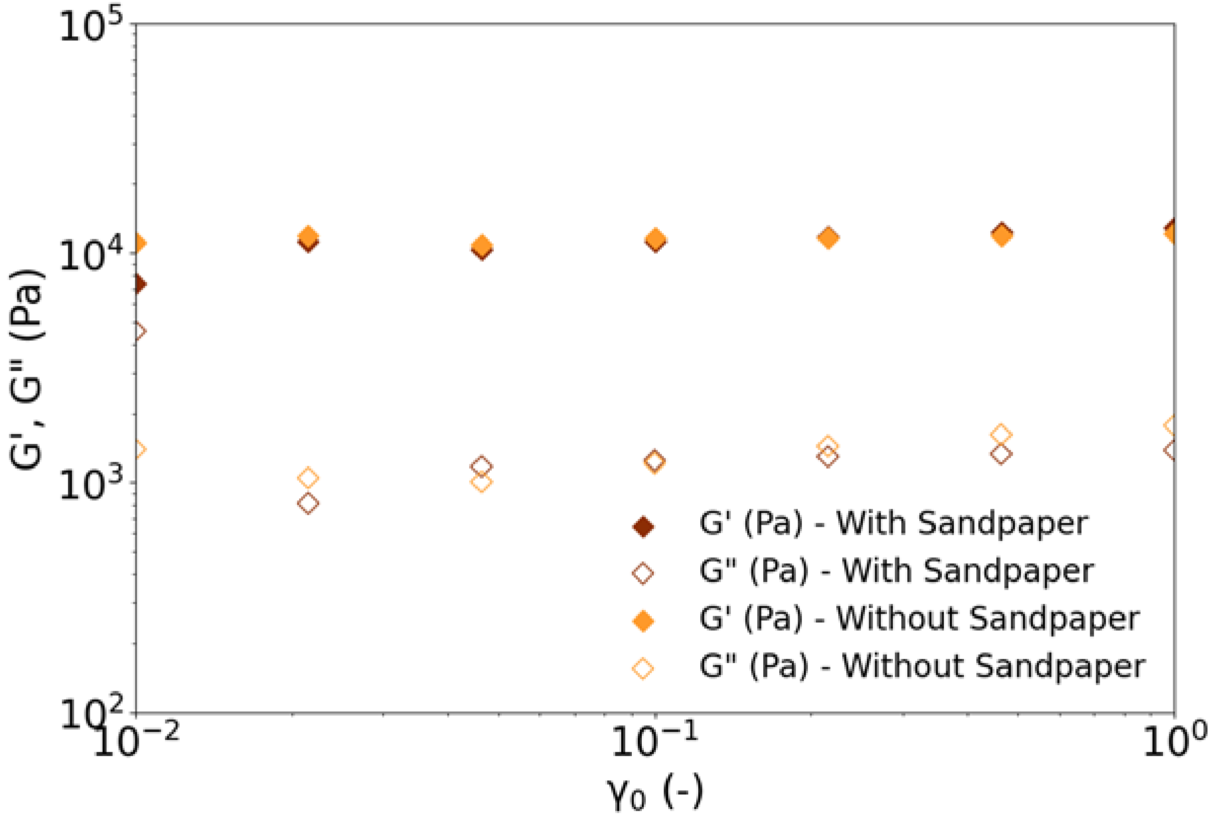

FIG. S2. Comparing frequency spectra of Alg+/PAAm+ hydrogel tested without sandpaper to with sandpaper. No considerable difference in frequency sweep was noted.

S3. ZERO AMPLITUDE TANGENT SLOPE G'_M AND LARGE AMPLITUDE SECANT SLOPE G'_L

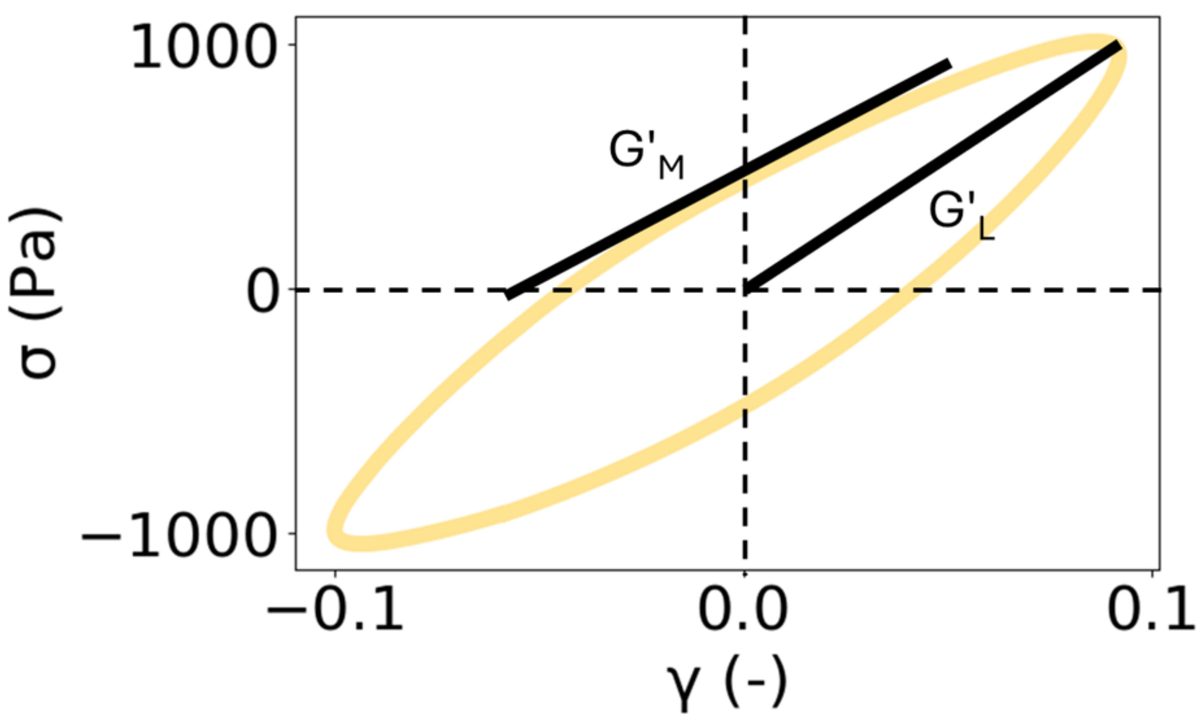

FIG. S3. Demonstration of zero amplitude tangent slope G'_M and large amplitude secant slope G'_L.

S4. CHEBYSHEV ELASTIC COEFFICIENTS FOR ALL THE HYDROGEL DESIGNS

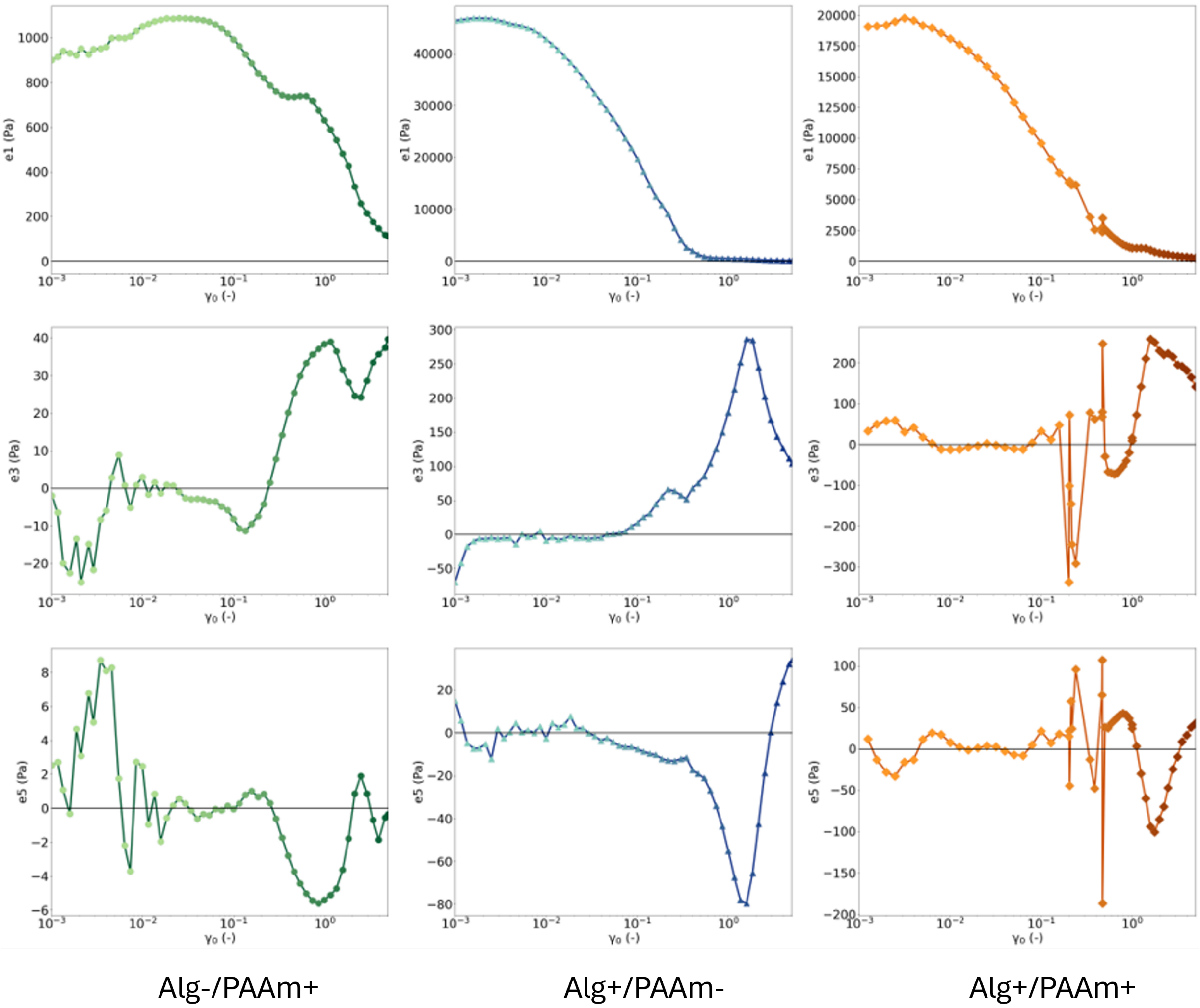

FIG. S4. Representation of e_1, e_3, and e_5 for all the hydrogel designs. The non-linearities arise in form of e_3 deviating from 0 as γ_0 goes beyond linear viscoelastic regime. At high γ_0, e_3 exhibits positive values highlighting primarily intracycle strain stiffening behaviour of the hydrogels. The initial non-zero e_3 and e_5 values at low γ_0 can be attributed to the rheometer's insensitivity to low torque as preference was given position sensitivity for non-linear measurements.

S5. ELASTIC STRESS EVOLUTION WITH OSCILLATORY CYCLES INCREASING AMPLITUDE

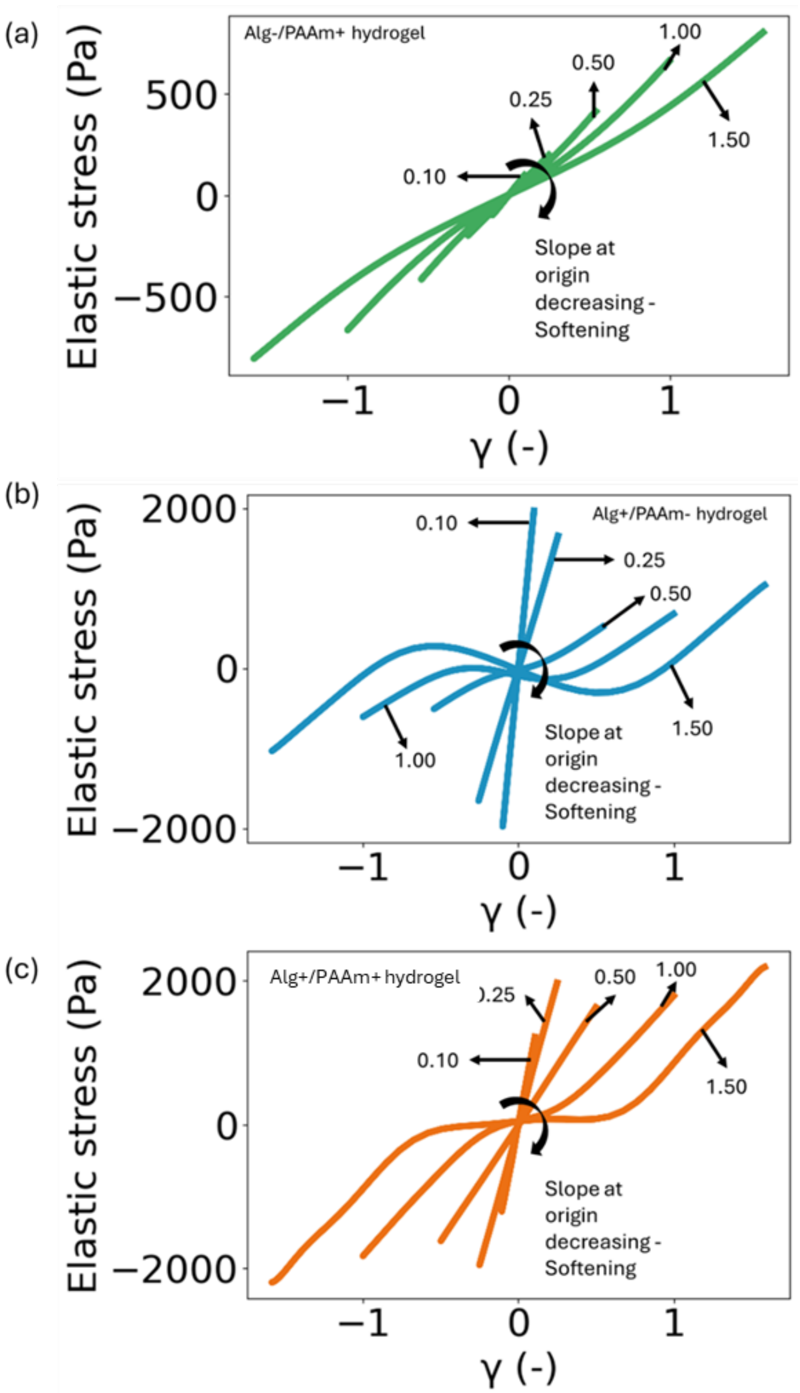

FIG. S5. Elastic stress evolution of hydrogels. (a) Alg-/PAAm+ hydrogel (b) Alg+/PAAm- hydrogel (c) Alg+/PAAm+ hydrogel. All hydrogels exhibit softening as the slope of elastic stress vs strain decreases with increasing amplitude of oscillatory cycles.

S6. FORWARD AND REVERSE AMPLITUDE SWEEP OF ALG-/PAAM$_{H}YDROGEL$

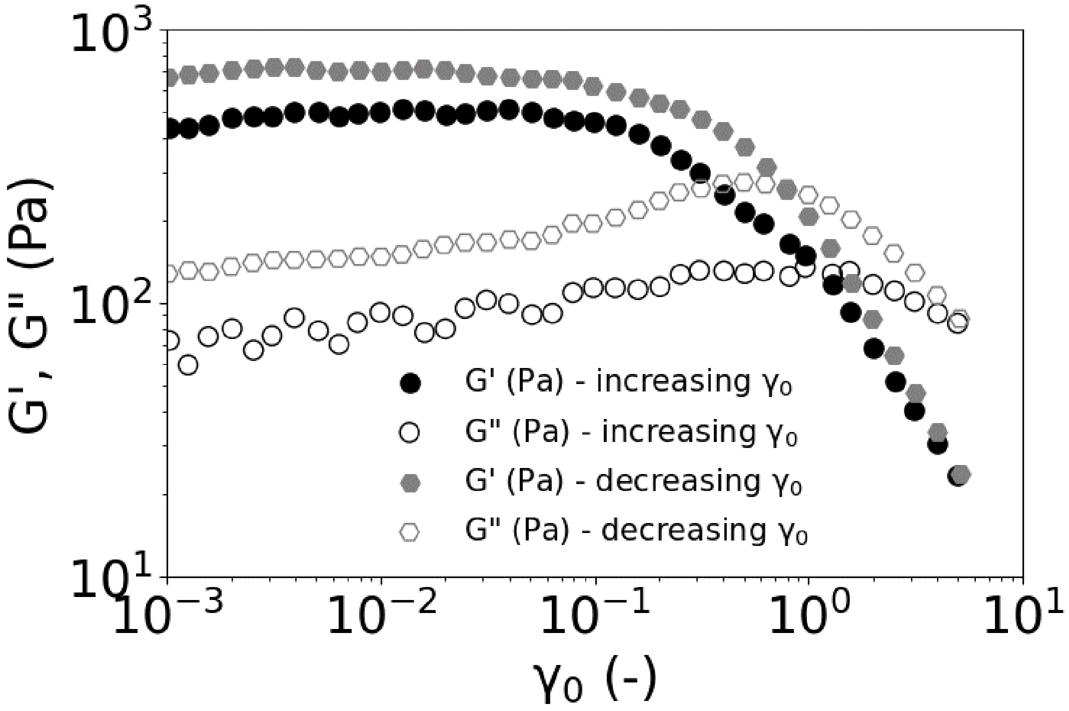

FIG. S6. Forward (increasing γ_0) and reverse (decreasing (γ_0)) amplitude sweeps demonstrates that the majority of the acrylamide network structure remains intact. The final values of G′ and G′′ in reverse amplitude sweep are slightly higher than the forward amplitude sweep which could potentially be due to the polymer chains aligning in the direction of shear during the forward amplitude sweeps leading to slightly stiffer network.

S7. COMPARISON BETWEEN ELASTIC MODULUS AND CAGE MODULUS IN LVE REGIME

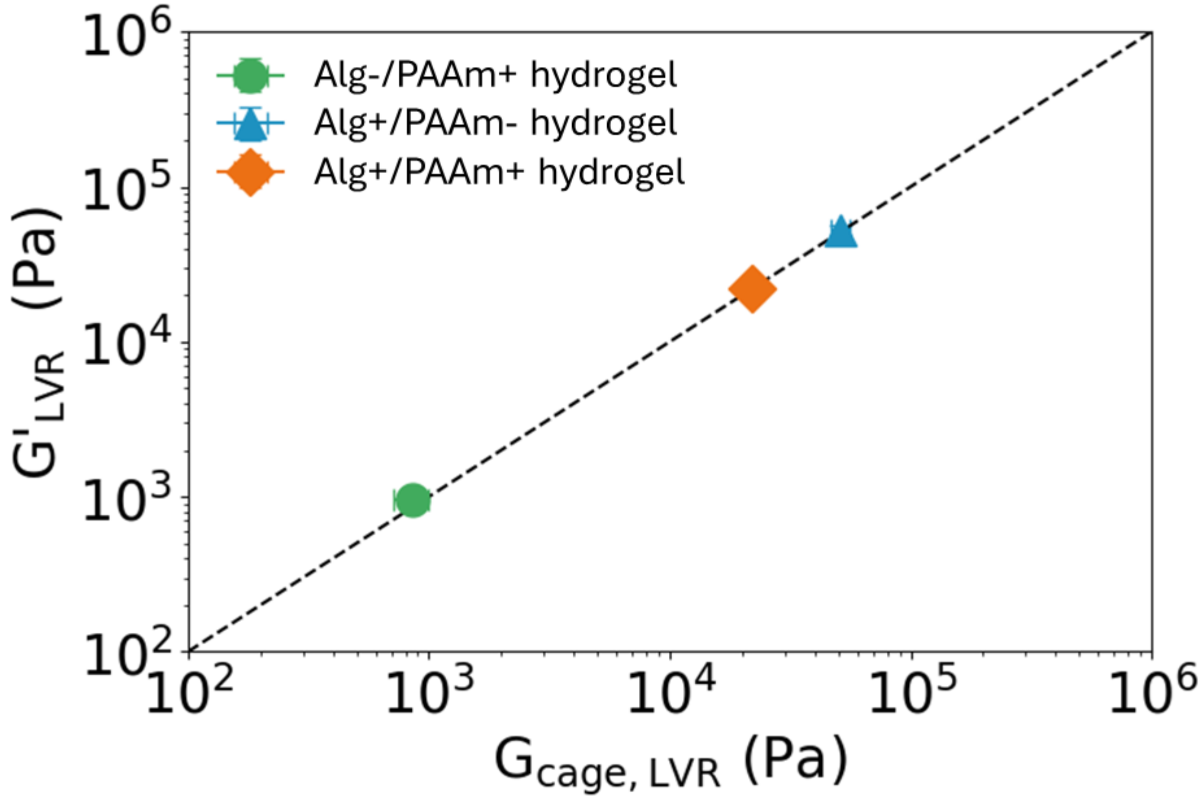

FIG. S7. Comparison between storage modulus and cage modulus in LVE regime. (a) Alg-/PAAm+ hydrogel (b) Alg+/PAAm- hydrogel (c) Alg+/PAAm+ hydrogel. The representative dashed line represents the line on which elastic modulus and cage modulus will be equal.

S8. COMPARISON OF PEAKS IN ELASTIC CONTRIBUTION TO STRESS AND S

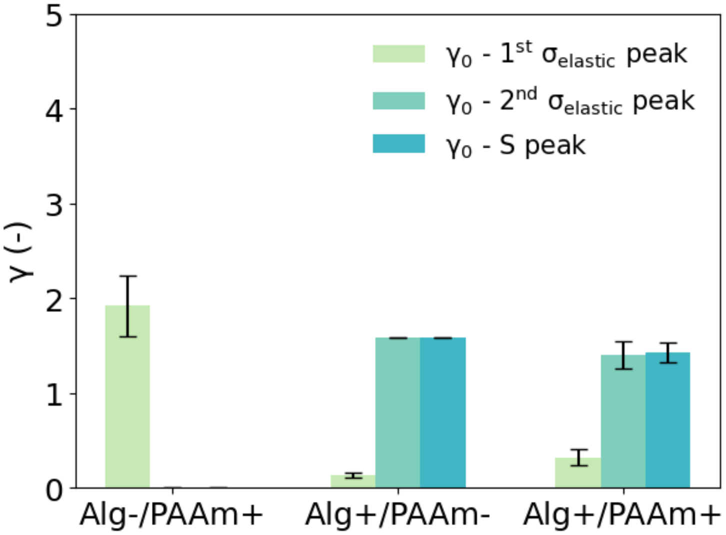

FIG. S8. Comparison of peaks in elastic contribution to stress and S (a) Alg-/PAAm+ hydrogel (b) Alg+/PAAm- hydrogel (c) Alg+/PAAm+ hydrogel.

S9. EVOLUTION OF TOTAL STRESS AND ITS ELASTIC AND VISCOUS STRESS COMPONENTS

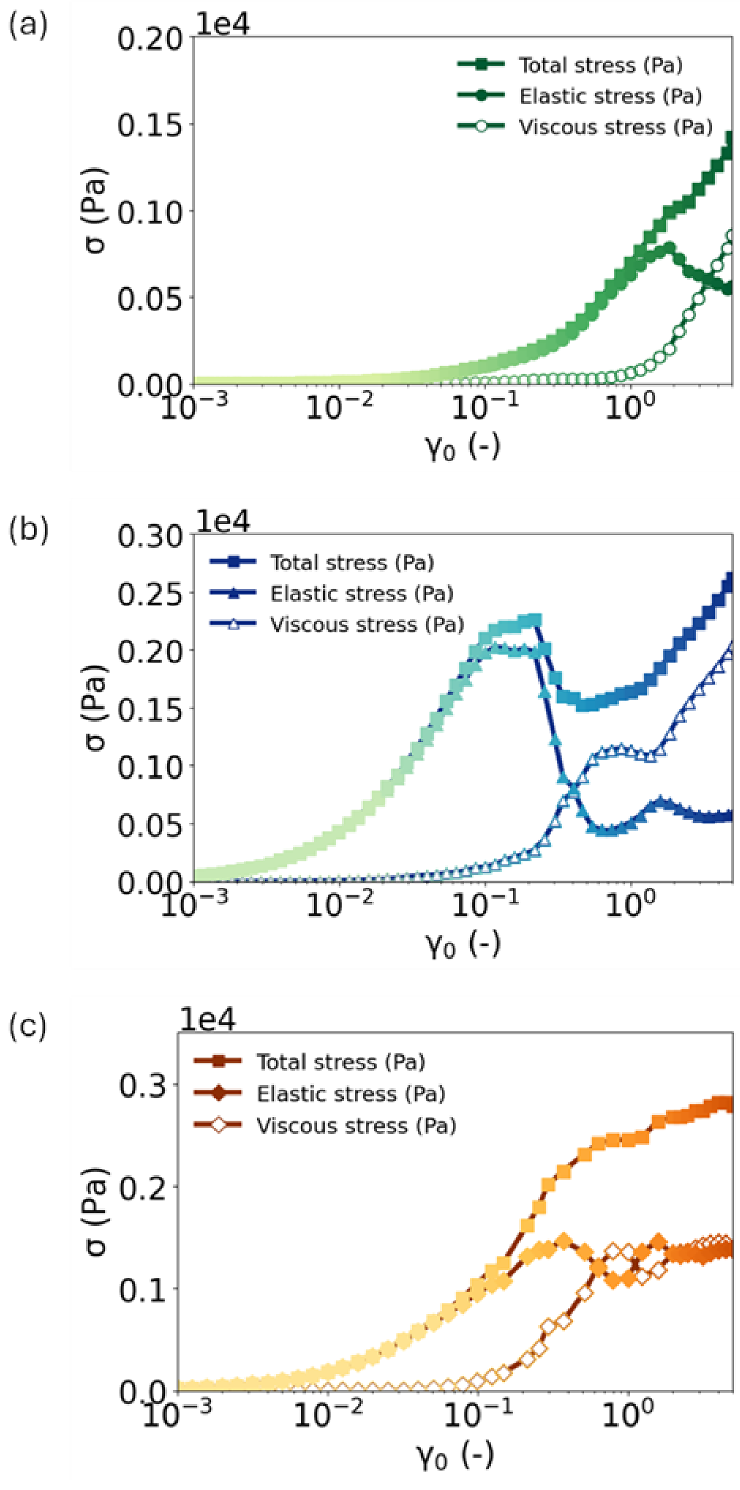

FIG. S9. Evolution of total stress and its elastic and viscous stress components. (a) Alg-/PAAm+ hydrogel (b) Alg+/PAAm- hydrogel (c) Alg+/PAAm+ hydrogel.

S10. COMPARISON OF G′, G′′, AND ELASTIC STRESS CONTRIBUTION AT THE FIRST PEAK FOR DIFFERENT AGENTS

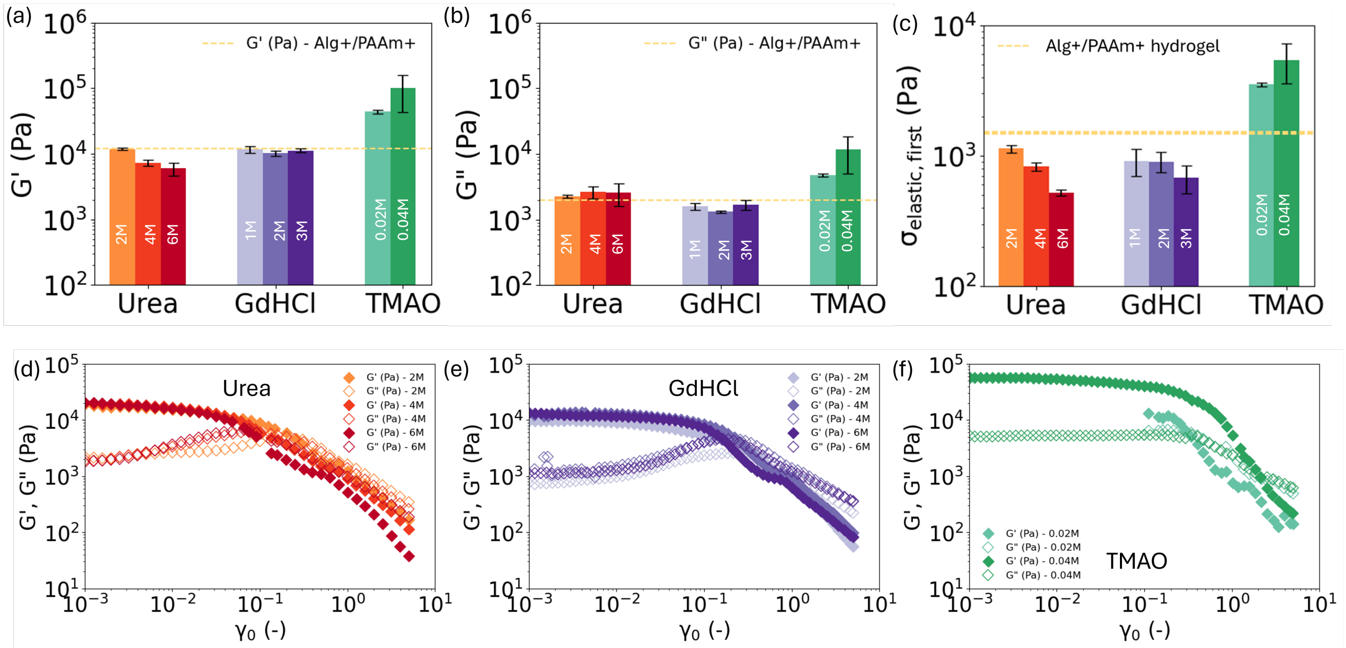

FIG. S10. (a) G′ and (b) G′′ of hydrogels soaked in different agents in LVE regime as measured at 0.5 Hz; (c) Comparison of elastic stress contribution at the first peak for various hydrogen bond strength tuning agents. The figure shows that the reduction in the hydrogen bonds results in dip in the additional peak of elastic stress contribution for urea and GdHCl. The effect is reversed when the hydrogen bonds are strengthened by the addition of TMAO; (d), (e), and (f) show the evolution of G′ and G′′ for Alg+/PAAm+ hydrogels treated with urea, GdHCl, and TMAO respectively. When treated with TMAO having concentration of 0.02M, the data below $\gamma_0 < 0.1$ is lost due to poor sensitivity of the rheometer to low torque.
